# Retrospective study of clinically relevant maxillary and mandibular anatomy in all skull types of dogs and cats

**DOI:** 10.3389/fvets.2025.1665996

**Published:** 2025-11-21

**Authors:** Erinn Schellenberg, Candace Lowe

**Affiliations:** Small Animal Dentistry and Oromaxillofacial Surgery, Small Animal Clinical Sciences, Western College of Veterinary Medicine, University of Saskatchewan, Saskatoon, SK, Canada

**Keywords:** ocular trauma, veterinary, cat, dog, infraorbital, dental, maxilla, mandible

## Abstract

**Introduction:**

In this retrospective study, computed tomography (CT) imaging was used to examine skull types of cats and dogs to provide clinically useful information for improved patient safety, regarding ocular trauma and hemorrhage control during dental nerve block administration, extraction of the caudal maxillary teeth, and caudal maxillectomy or mandibulectomy.

**Materials and methods:**

CT imaging was used to examine the images of 193 dogs and 41 cats, which were divided into mesocephalic (≤5, 6–10, and ≥11 kg), brachycephalic (≤10 and ≥11 kg), dolichocephalic (≤10 and ≥11 kg) dogs, and mesocephalic and brachycephalic cats. Descriptive statistics were calculated for infraorbital canal length, width and height, shortest distances for infraorbital foramen to globe, maxillary foramen to globe, and maxillary first and second molar tooth root apex to globe in dogs, and apices of the maxillary fourth premolar tooth to globe in cats, palate to maxillary foramen, palate to globe, and mandibular molar to mandibular foramen in all cats and dogs. Values were tabulated and combined into reference materials to aid veterinarians in making safer clinical decisions.

**Results:**

The shortest maxillary molar tooth root apex-to-globe minimum value was 2.1mm in mesocephalic ≤5kg dogs. The shortest minimum palate-to-globe distance was 5.3mm in mesocephalic dogs of 6-10kg. The minimum maxillary fourth premolar tooth root apex-to-globe distances ranged from 1.6-2.8mm in all cats. Accidental globe puncture through the infraorbital canal was possible in 100% of cats and dolichocephalic ≤10kg dogs, and between 81-95% of mesocephalic dogs weighing 10kg or less.

**Conclusion:**

Caution should be taken when performing infraorbital and maxillary nerve blocks in mesocephalic and dolichocephalic dogs ≤10 kg and all cats. Using proper technique, brachycephalic ≥11kg dogs may be at lower risk of accidental globe trauma during infraorbital nerve block. The deep maxillary nerve block should not be used in cats or small dogs. The maxillary nerve block, using the modified infraorbital canal approach, combined with the author’s recommended safe needle/catheter insertion distances should provide safe, effective analgesia administration. Mean measurements for mandibular molar to mandibular foramen, and maxillary molar to maxillary foramen may be used for surgical planning and ligation for hemostasis for caudal mandibulectomies and maxillectomies, particularly when CT imaging is not available.

## Introduction

Dental disease is one of the most common conditions affecting canine and feline patients in veterinary medicine. Wide ranges in prevalence have been reported, with maximum values of 80–86% of canine and feline patients over 2–3 years of age affected (1, 2). Because of this, oral hygiene procedures with dental extractions are increasingly performed in small animal practice.

**Figure 1:**
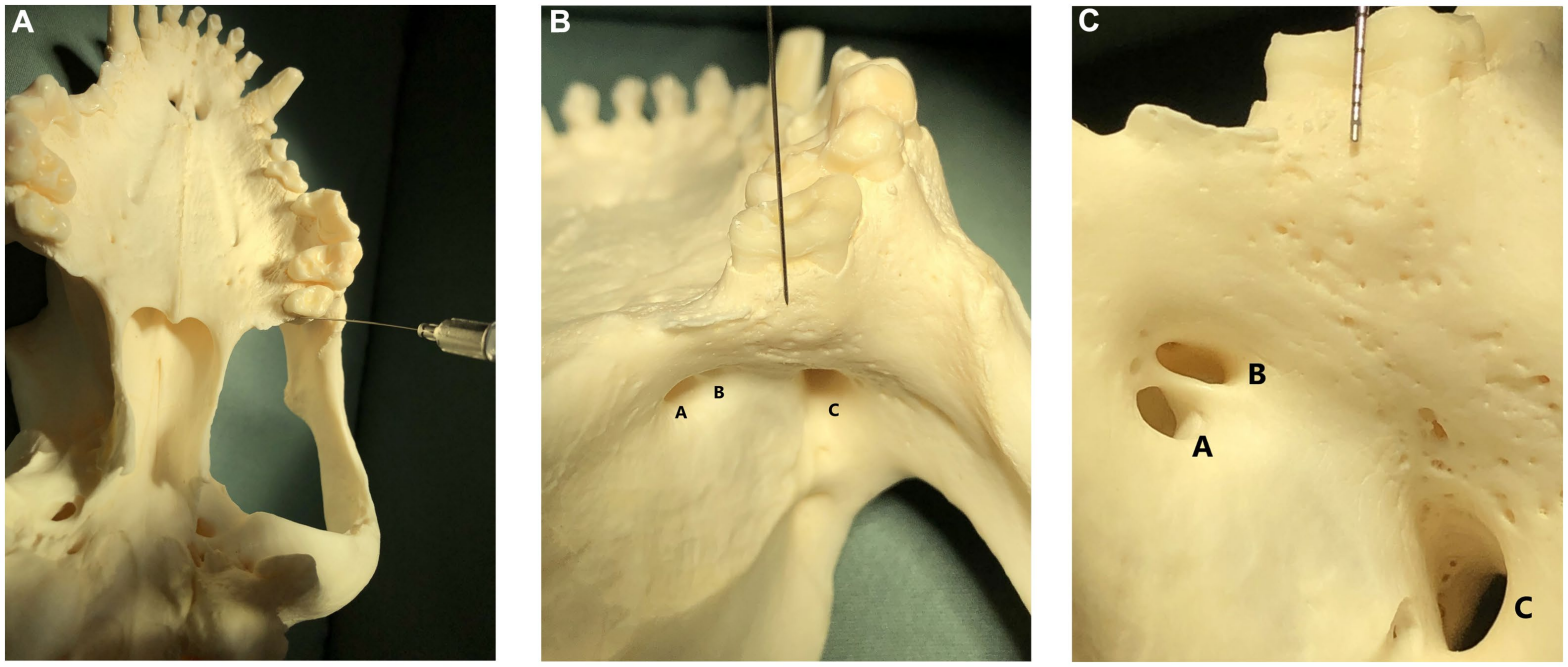
**(A)** Image showing a skull in dorsal recumbency with a nerve block needle pointing to the caudal border of the hard palate, immediately caudal to the left maxillary second molar tooth. This image is shown for orientation in subpart (C); **(B)** Image shown in subpart (A) is zoomed in 50% and shows a skull in dorsal recumbency with a nerve block needle pointing to the caudal border of the hard palate, immediately caudal to the left maxillary second molar tooth. This image is shown for orientation in subpart (C). The sphenoplatine (A), caudal palatine (B), and maxillary foramina (C) are labeled. **(C)** Image shown in subpart (B) is zoomed in 50% and shows a skull in dorsal recumbency. A dental probe is pointing to the caudal border of the hard palate, immediately caudal to the left maxillary second molar tooth. Multiple tiny foramina, where tiny nerves from the caudal branch of the superior alveolar nerve enter the bone to innervate the maxillary molar teeth, are visible beneath the dental probe as tiny holes in the bone. The sphenopalatine foramen (A) and caudal palatine foramen (B) are the two smaller foramina on the left. The maxillary foramen, or entrance to the infraorbital canal, is visible as the large foramina on the lower right (C).

**Figure 2:**
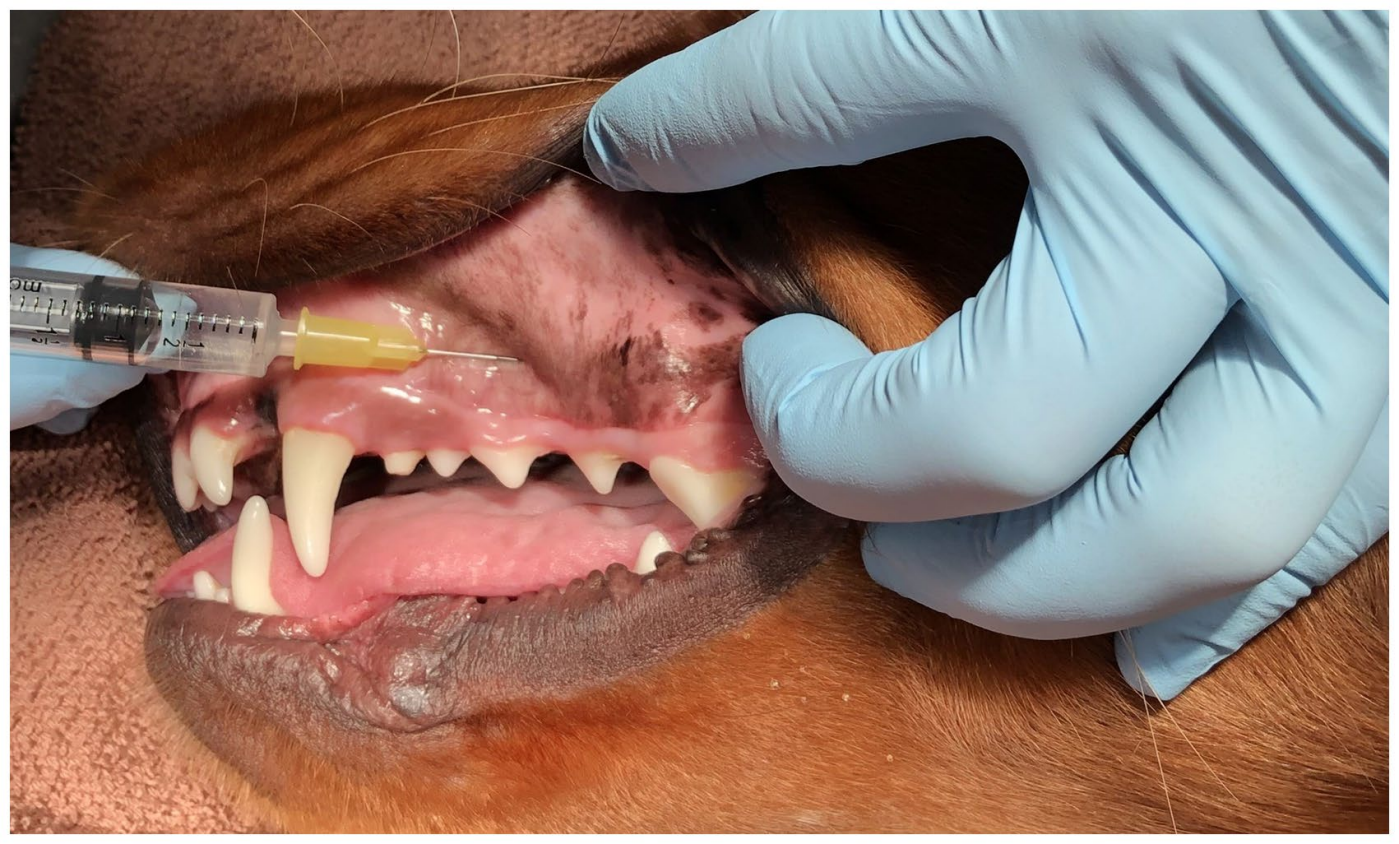
Image of a mesocephalic dog lying in right lateral recumbency for demonstration of the proper technique for infraorbital canal nerve block. Of crucial importance, notice that the length of the needle is exactly parallel to the hard palate and NOT angled upward to the eye.

Multimodal analgesic approaches utilizing infraorbital, maxillary, and mandibular nerve blocks are recommended for perioperative reduction in pain and improvement in postoperative comfort in small animal veterinary dentistry (3). Dental local nerve blocks provide analgesia and improve anesthetic management by significantly reducing the isoflurane minimum alveolar concentration of patients under general anesthesia (4, 5). Innervation to the maxillary teeth arises initially from the maxillary nerve, a subset of the trigeminal nerve (6). The maxillary nerve travels underneath the eye to become the infraorbital nerve after the caudal nasal nerve branches off in the pterygopalatine fossa (6). The infraorbital nerve sends branches off into the caudal, middle, and rostral superior alveolar nerves to innervate the maxillary teeth (6–8). The caudal branches, which innervate the molar teeth and sometimes part of the maxillary fourth premolar tooth in most dogs, exit the infraorbital nerve before entering the maxillary foramen of the infraorbital canal under the eye, as seen in Figures 1A–C (3, 6–8). The middle and rostral superior alveolar branches, located within the infraorbital canal, innervate the remaining maxillary teeth (3, 6, 8). The infraorbital nerve block provides adequate pain relief for maxillary extractions in all cats and most canine patients, with a few exceptions (3, 9–11). Pascoe revealed that the infraorbital nerve block did not consistently provide adequate analgesia for the last 3 maxillary teeth in some dogs (12). Because of this, proper analgesia in canine patients may require the maxillary block prior to painful stimuli of their maxillary molar tooth, sometimes their maxillary fourth premolar tooth, and patients with tumors, fractures or other conditions where the infraorbital block is contraindicated or has provided inadequate analgesia. In cats, the infraorbital block consistently provides analgesia for all maxillary teeth, except the maxillary first molar tooth (9). Due to the increased risk of ocular trauma in feline patients, the maxillary block is deemed unnecessary (9). There are multiple reported cases of accidental ocular trauma during administration of the maxillary nerve block in cats and dogs (9, 10, 13–15). In our practice, the infraorbital block is adequate most of the time for all patients. There are at least five reported approaches to performing the maxillary nerve block in dogs and cats (1, 3, 10, 16, 17). The caudal intraoral approach to maxillary nerve block has high potential for ocular trauma if done incorrectly and can be done in one of two ways (3, 13). In both methods, the needle is inserted into the mucosa immediately behind the last maxillary molar tooth (maxillary second molar tooth in dogs and maxillary first molar tooth in cats). With the deep approach, the needle is inserted into the pterygopalatine fossa, at the caudal entrance to the infraorbital canal, otherwise known as the maxillary foramen, to anesthetize the maxillary nerve (3). To prevent trauma while performing the infraorbital nerve block, needles and catheters should always be inserted into the infraorbital canal parallel to the hard palate (Figure 2) and should never be angled upward because the upward angulation shortens the distance to globe penetration. Knowledge of regional anatomy can significantly reduce or prevent the incidence of intraocular trauma and improve the efficiency of surgical technique, while also playing a role in the patient’s multimodal analgesia. There are at least nine reports in the literature of penetrating eye injury from either the infraorbital nerve block (18–20), maxillary nerve block (9, 13, 14), or from dental extractions in the maxillary arcade (13, 15, 21, 22). Recent literature has shown improvements in surgical techniques for advanced procedures involving the caudal mandible and maxilla, aiming to enhance patient safety and reduce technical difficulty (7, 23). Computed tomography (CT) imaging has been used to retrospectively describe the specific anatomy of the infraorbital canal in mesocephalic and brachycephalic cats (24). To the author’s knowledge, similar studies have not been performed in dogs.

The objective of this study was to describe clinically useful skull anatomy that will aid in improved ocular safety, clinician efficacy when performing dental nerve blocks and extractions, and efficiency when performing dental extractions, caudal mandibulectomies, and maxillectomies in the cat and dog using established weight and skull type categories. Specifically, the objective of this study was to use CT imaging to retrospectively describe, for mesocephalic, brachycephalic and dolichocephalic dogs of various body weights, and for mesocephalic and brachycephalic cats, the infraorbital canal, globe diameter, the shortest distance from the infraorbital foramen to the globe, the shortest distance from apices of the maxillary first and second molar teeth to the globe in dogs and maxillary fourth premolar tooth to the globe in cats, length of the distal buccal root of the maxillary first and second molar teeth in dogs, the distance from the caudal palate-to-globe, the distance from the caudal palate-to-maxillary foramen and mandibular third molar tooth in dogs and mandibular first molar tooth in cats, to the mandibular foramen in all dogs and cats.

## Materials and methods

**Figure 3:**
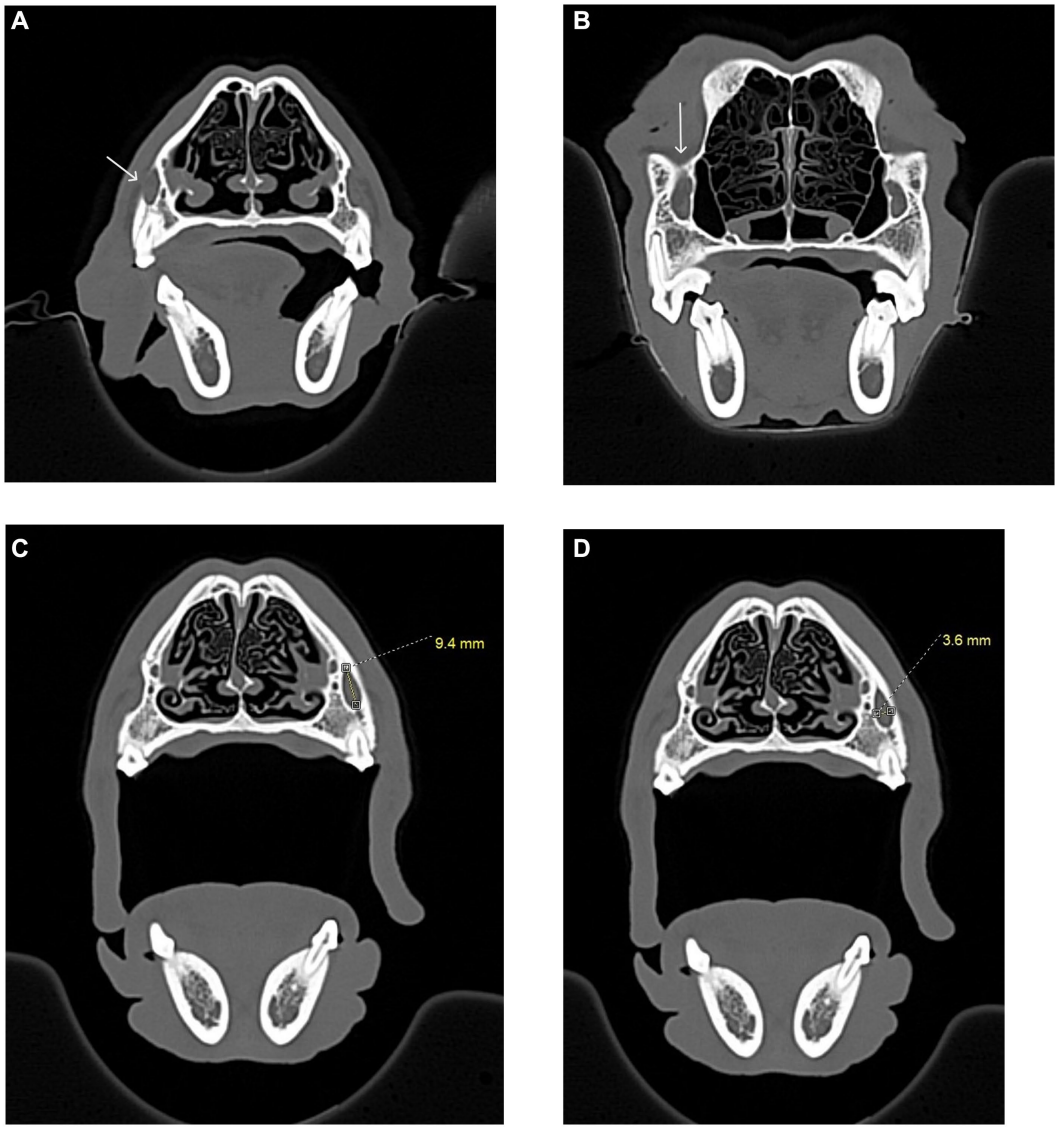
Infraorbital canal length measurement, in millimeters, was taken using the CT transverse view, from the infraorbital foramen to the maxillary foramen in the cat and dog. **(A)** The white arrow shows the right infraorbital foramen in a dog skull. A complete bony circle is barely visible. **(B)** The white arrow shows the right maxillary foramen in a dog skull. A complete bony circle is visible. **(C)** Image showing the height measurement of a dog’s left infraorbital canal. The measurement was collected at the infraorbital foramen. **(D)** Image showing the width measurement of a dog’s left infraorbital canal. The measurement was collected at the infraorbital foramen.

**Figure 4:**
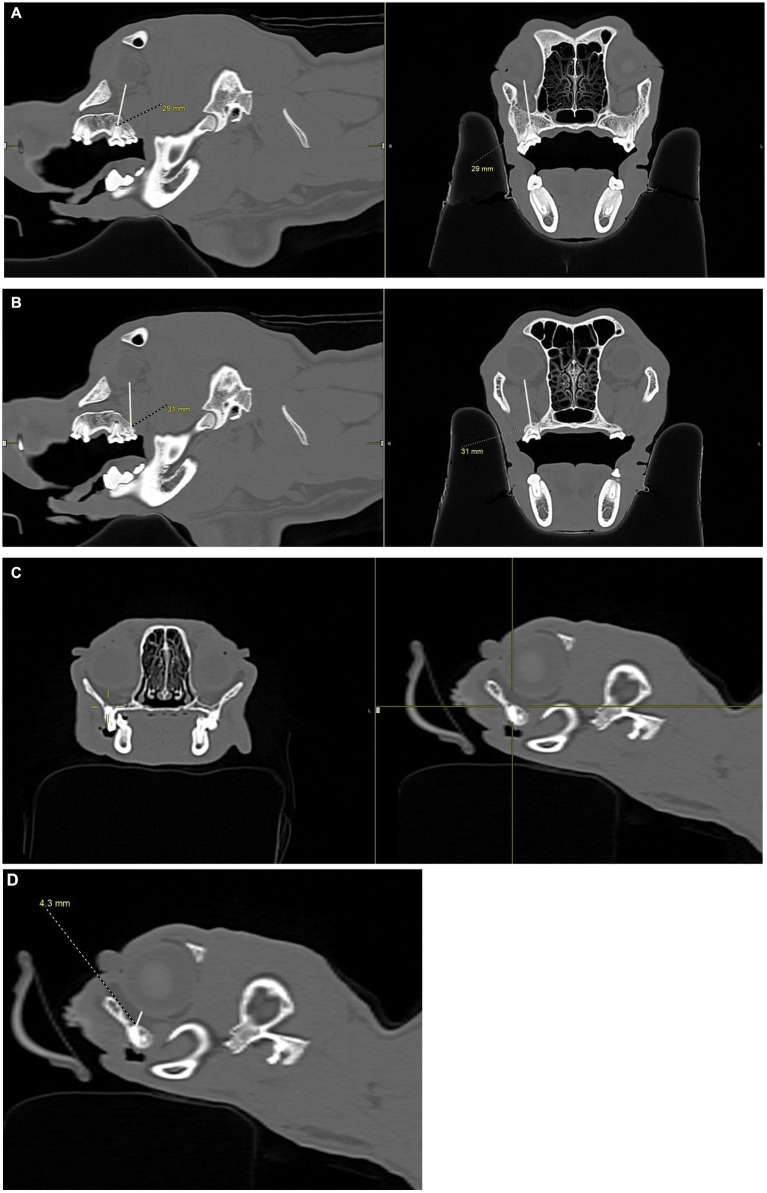
CT images showing how measurements, in millimeters, were collected from the apices of the maxillary first molar tooth and second molar tooth to the globe in dogs, and from the maxillary fourth premolar tooth to the globe in cats. **(A)** Distance from the right maxillary first molar tooth palatal root apex to the globe using both the sagittal (left) and transverse (right) views simultaneously for accuracy. **(B)** Distance from the right maxillary second molar tooth distal buccal root apex to the globe using both the sagittal (left) and transverse (right) views simultaneously for accuracy. **(C)** Transverse (left) and sagittal (right) views were used to confirm the right maxillary fourth premolar tooth distal root apex. The root apex is visible in the crosshairs on both the transverse and sagittal views. **(D)** After confirming the right maxillary fourth premolar tooth distal root apex **(C)**, the distance from the root apex to the closest surface of the globe was measured.

**Figure 5:**
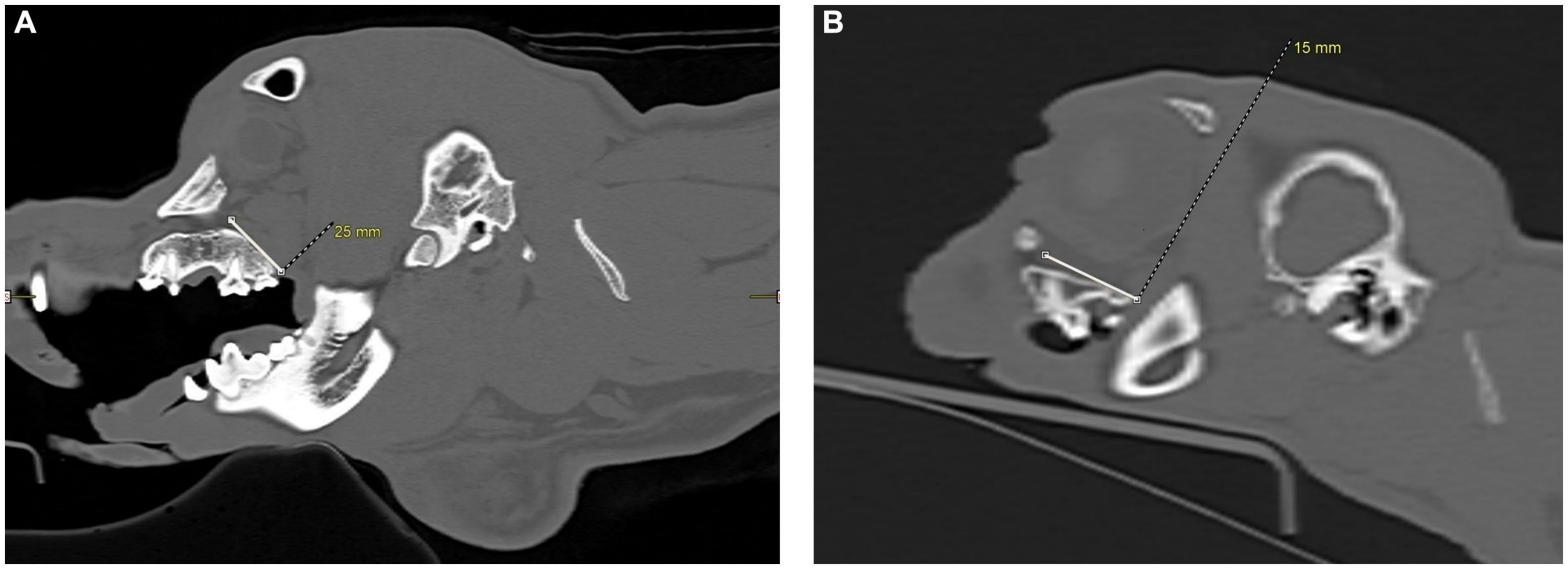
CT images showing measurements, in millimeters, taken from the caudal hard palate, immediately caudal to the maxillary second molar tooth in dogs and maxillary first molar tooth in cats, to the maxillary foramen. **(A)** Sagittal view of a dog skull showing the measurement from the caudal palate, immediately caudal to the maxillary second molar tooth, to the maxillary foramen. **(B)** Sagittal view of a cat skull showing the measurement, in millimeters, from the caudal palate, immediately caudal to the maxillary first molar tooth, to the maxillary foramen.

**Figure 6:**
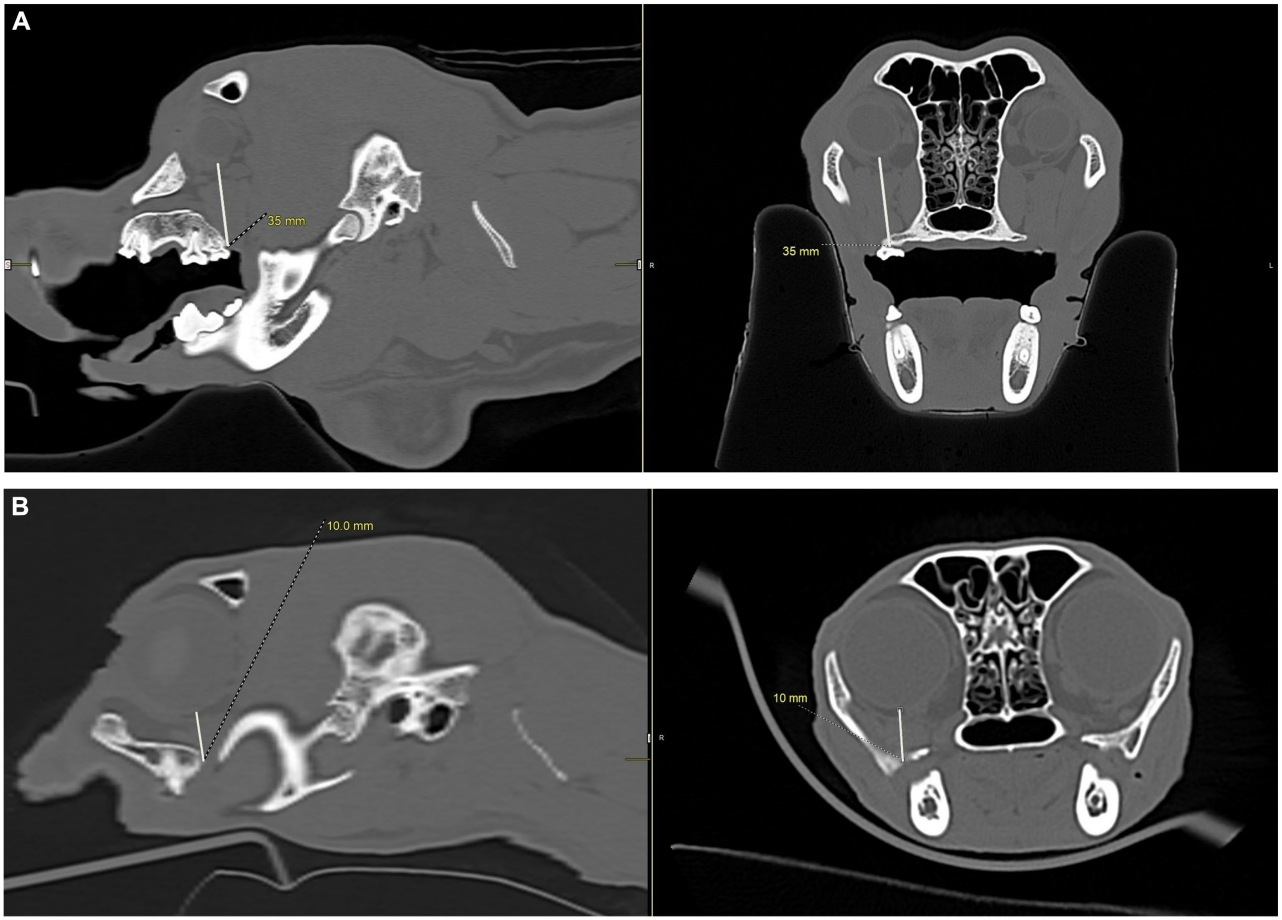
CT images showing measurements, in millimeters, from the caudal palate, immediately caudal to the maxillary second molar tooth in dogs and the maxillary first molar tooth in cats, to the closest surface of the globe. **(A)** Sagittal (left) and transverse (right) views of a dog skull showing the measurements taken immediately caudal to the right maxillary second molar tooth to the closest surface of the globe. **(B)** Sagittal (left) and transverse (right) views of a cat skull showing the measurements taken immediately caudal to the right maxillary first molar tooth to the closest surface of the globe.

**Figure 7:**
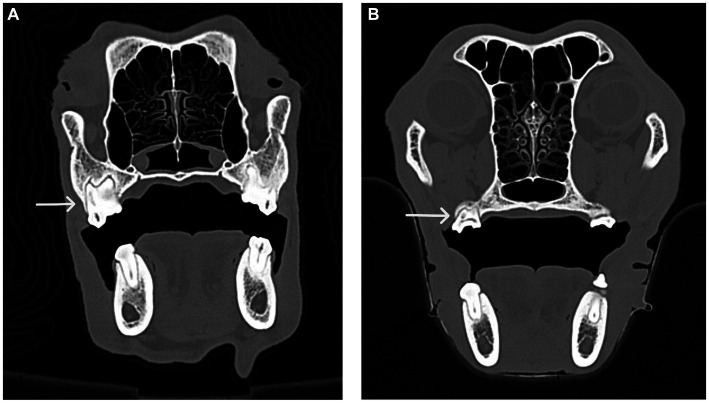
CT images of the transverse view of dog skulls showing the fusion of roots in the maxillary first molar tooth and the maxillary second molar tooth. **(A)** This CT image shows fusion (white arrow) of the distal buccal root with the palatal root of the maxillary first right molar tooth in a dog. **(B)** This CT image shows fusion (white arrow) of the distal buccal root with the palatal root of the right maxillary second molar tooth in a dog.

**Figure 8:**
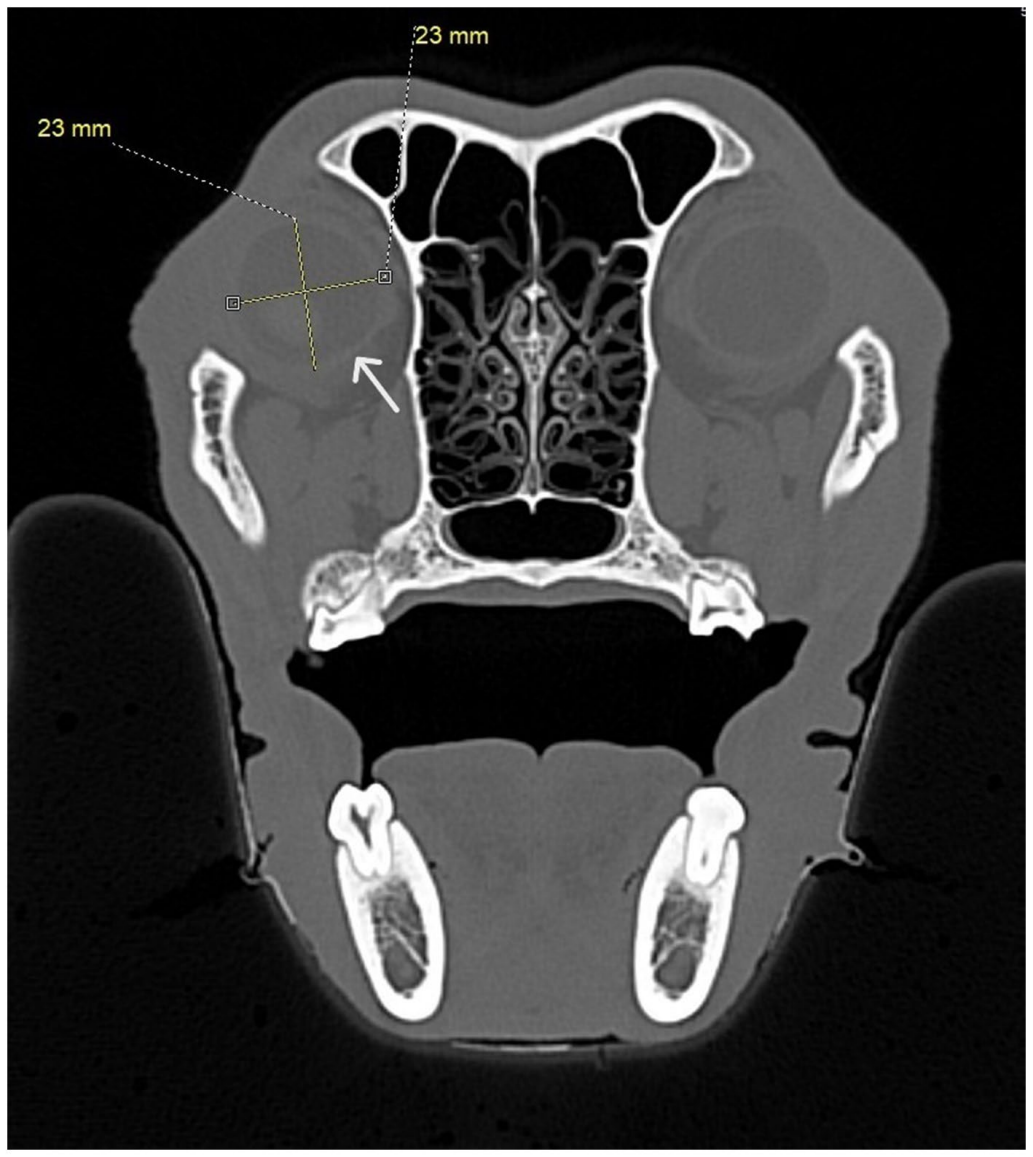
Transverse CT image showing diameter measurements taken for each eye. At least four measurements were taken at right angles to one another. The white arrow shows the border of the eye.

**Figure 9:**
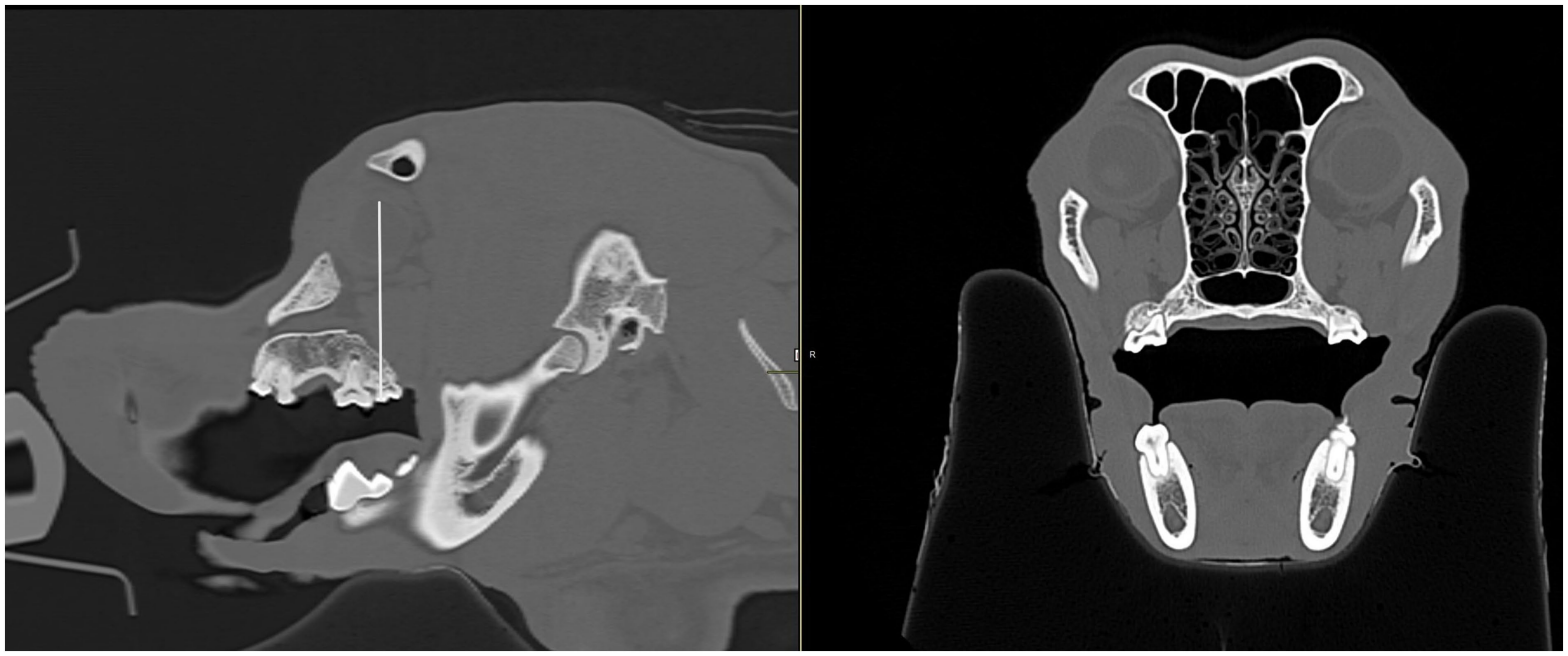
Sagittal and transverse CT images of a dog skull. Using the sagittal view (left), a line was drawn through the center of the globe and continued through the corresponding maxillary tooth immediately below to determine which tooth the eye was centered over. The transverse view (right) was then used to confirm the maxillary tooth.

**Figure 10:**
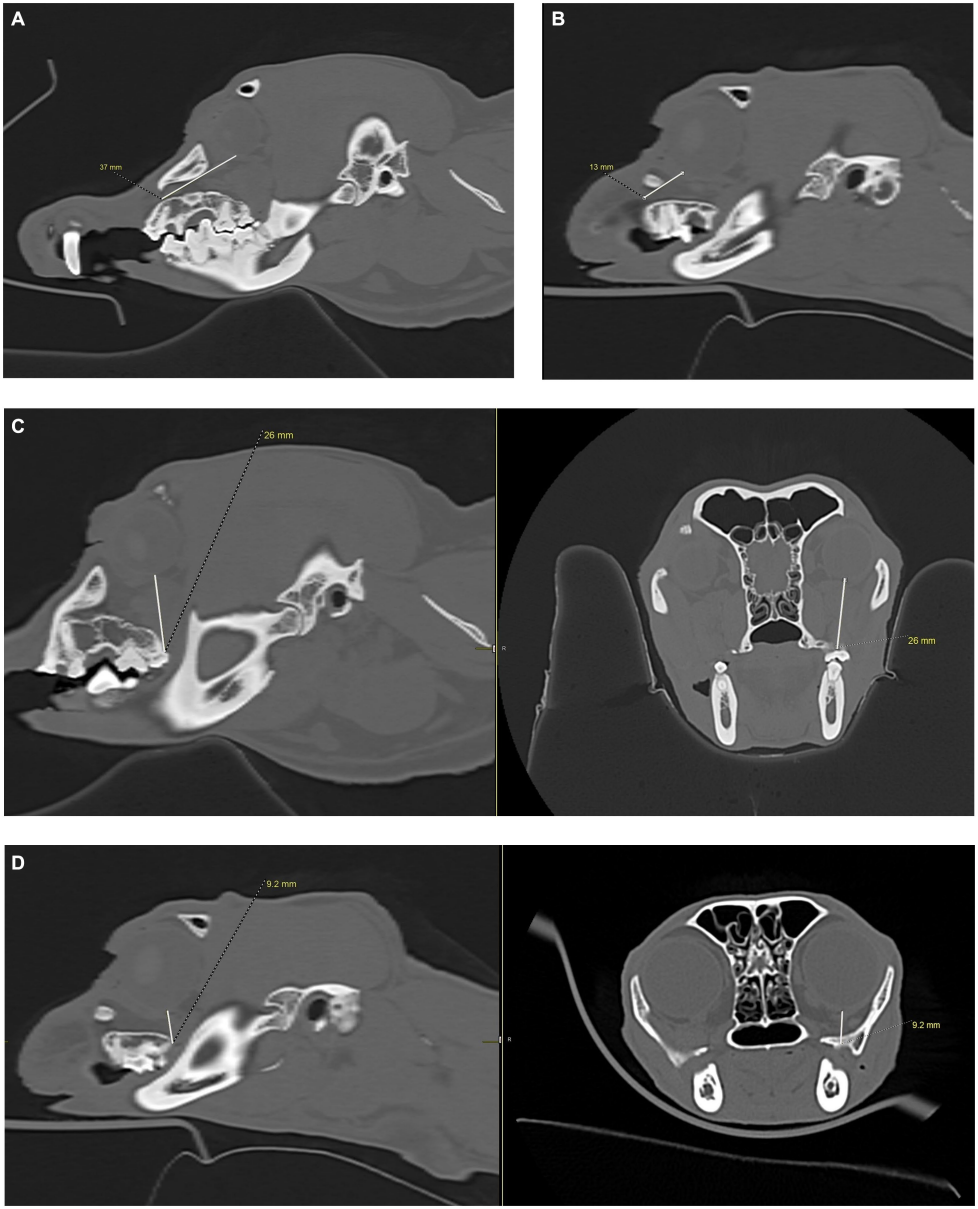
CT images showing measurements from the infraorbital foramen-to-globe and hard palate-to-globe distances in the cat and dog. Measurements were taken to simulate accidental globe puncture during an infraorbital nerve block **(A,B)**, maxillary nerve block using the modified infraorbital approach **(A,B)**, and the caudal maxillary nerve block **(C,D)**. **(A)** This sagittal CT image shows the measurement, in millimeters, from the infraorbital foramen to the closest surface of the globe (white line) in a dog. **(B)** This sagittal CT image shows the measurement, in millimeters, from the infraorbital foramen to the closest surface of the globe (white line) in a cat. **(C)** Sagittal (left) and transverse (right) CT images of a dog skull showing the measurement, in millimeters, from the caudal border of the palate, immediately caudal to the left maxillary second molar tooth, to the closest surface of the globe (white line). **(D)** Sagittal (left) and transverse (right) CT images of a cat skull showing the measurement, in millimeters, from the caudal border of the palate, immediately caudal to the left maxillary first molar tooth, to the closest surface of the globe (white line).

CT images from the University of Saskatchewan’s Veterinary Medical Center (VMC) Picture Archive System (PACS) were examined retrospectively. All clients of the VMC sign a research release upon admission of their animal to the hospital. Standard and PET CT head scans, performed between 1 January 2010 and 1 July 2025, were evaluated for inclusion in the study. All patients included in this study were scanned in sternal recumbency. Multiplanar reformatting was previously performed on scans that were not straight as per the general hospital CT scanning protocol. All images were 1 mm slices or smaller using standard CT imaging, except 8 mesocephalic dogs, which used 1.25 mm slice images from the PET CT, and 2 ≥11 kg dolichocephalic dogs (one at 1.25 mm and one at 2 mm slices) from the PET CT. CT images were examined until 32 patients had been reached in a category, or until all patients of the size and category had been exhausted in the PACS system. Initially, only patients with full caudal maxillary and caudal mandibular dentition were included. Once these patients had been exhausted, all CT images within the date range were re-examined, and patients with the most complete dentition were included. All measurements were taken using two-dimensional (2D) transverse and sagittal views. CT images of 193 dogs and 41 cats were included in the study. Animals were excluded if local anatomy was distorted or if the CT slices were greater than 1.25 mm. Dogs were separated into mesocephalic, brachycephalic, and dolichocephalic skull types based on skull index categories described by Ichikawa et al. (25). The canine skull index was calculated for mixed-breed dogs to confirm which category to place them in. Dogs with a canine skull index less than 51 were considered dolichocephalic. Dogs with a canine cephalic index between 51 and 59 were considered mesocephalic. Dogs with a canine cephalic index greater than 59 were considered brachycephalic (25). Canine patients were further subdivided based on body weight. There were only two brachycephalic and three dolichocephalic dogs weighing less than 5 kg that met the inclusion criteria for the 15-year date range of this study. Conversely, more than 32 mesocephalic dogs weighing less than 5 kg were available for inclusion in this study. Clinically, the authors have noted that ≤5 kg mesocephalic dogs are frequently presented for oral hygiene procedures. In the author’s opinion, mesocephalic dogs weighing less than 5 kg are more technically challenging to perform procedures on due to the small size of their oral cavity. For these reasons, brachycephalic and dolichocephalic dogs were separated into two weight categories, and mesocephalic dogs were separated into three weight categories. Dolichocephalic dogs were divided into large and small body weight groups, namely, ≥11 and ≤10 kg, respectively. Brachycephalic dogs were divided into large and small body weight groups, namely, ≥11 and ≤10 kg, respectively. Mesocephalic dogs were divided into large, small, and extra small body weight groups, due to the prevalence of extra small dogs, namely, ≥11, 6–10, and ≤5 kg, respectively. Feline patients were placed into mesocephalic (MCat) and brachycephalic (BCat) groups based on skull type only. Body weight was not used to place feline patients in different categories. In total, the dolichocephalic dogs group consisted of 22 dogs ≥11 kg and 15 dogs ≤10 kg. The mesocephalic dogs group consisted of 32 dogs ≤5 kg, 30 dogs of 6–10 kg, and 32 dogs ≥11 kg. The brachycephalic dogs group consisted of 31 dogs ≥11 kg and 31 dogs ≤10 kg. A total of 31 mesocephalic and 10 brachycephalic cats were included. Signalment data, including breed, sex, age, bodyweight, and diagnosis, were recorded for each patient. Descriptive statistics were performed in the analysis. Mean, minimum, maximum, median, mode, and standard deviation (SD) were calculated for all data points and recorded in a Microsoft Excel spreadsheet. All measurements were recorded by the same person (E. Schellenberg) using the PACS system on the same high-resolution monitor available in the VMC radiology service. All patient selection was performed by the same person (E. Schellenberg). Measurements collected included infraorbital canal length, width, and height. Infraorbital canal length was assessed by measuring, in millimeters, on the transverse view moving rostral to caudal from the infraorbital foramen to the maxillary foramen. The infraorbital foramen was determined to be the first transverse slice where a complete bony circle was formed (Figure 3A). The maxillary foramen was determined to be the last transverse slice where a complete bony circle was formed (Figure 3B). Width and height were measured at the infraorbital foramen (Figures 3C,D). The shortest distance from the maxillary foramen to the closest outer surface of the globe was recorded on the sagittal view. In dogs, values were recorded for the shortest distance from root apices of the left and right maxillary first and second molar teeth to the globe (root apex-to-globe), using both transverse and sagittal views (Figures 4A,B). The shortest distance was almost always from the palatal root to the globe for the maxillary first molar tooth and varied between the buccal roots and the palatal root for the maxillary second molar tooth. In cats, the shortest root apex-to-globe distance was recorded, using the transverse and sagittal views, for the distal, mesial palatal, and mesial buccal roots of the left and right maxillary fourth premolar tooth (Figures 4C,D). In cats and dogs, the distance from the end of the hard palate, immediately caudal to the maxillary second molar tooth in dogs and maxillary first molar tooth in cats, to the maxillary foramen (palate-to-maxillary foramen) was recorded using the sagittal view (Figures 5A,B). Values were excluded if the maxillary second molar tooth in dogs or the maxillary first molar tooth in cats was absent, or in cases where the palatal bone appeared to be decreased in length. In cats and dogs, the shortest distance from the caudal border of the palate, immediately caudal to the maxillary second molar tooth in dogs and maxillary first molar tooth in cats, to the globe (palate-to-globe) was recorded using both the transverse and sagittal views (Figures 6A,B). In all dogs, the maxillary first and second molar teeth were assessed for fusion of the distal buccal root with the palatal root, and the results were recorded as a percentage (Figures 7A,B). There were no instances of the mesial buccal root appearing to be fused with the palatal root. The number of cats and dogs with bifurcation of the infraorbital canal was recorded for each skull and weight category. The average globe diameter was recorded for each animal on the transverse view using the largest diameter recorded that was repeatable at least 4 times with multiple right angles to each other, for each eye (Figure 8). The border of the eye was determined to be the outside edge of the smooth, round, radiopaque circle (Figure 8). To determine which tooth the globe was centered over, a vertical line was drawn down the center of each eye on the sagittal view, through the corresponding maxillary tooth immediately below, and then confirmed on the transverse view (Figure 9). Frequently, the globe center was close to two teeth. Only the most central tooth was recorded. For accuracy, the globe center was not recorded for animals that were missing teeth in that region. To simulate accidental globe puncture during the infraorbital nerve block and maxillary nerve block using the modified infraorbital approach, the shortest distance from the infraorbital foramen to the closest surface of the globe (infraorbital foramen-to-globe) was recorded, using the sagittal view, for all cats and dogs (Figures 10A,B). If accidental globe puncture was not possible due to the location of the eye relative to the infraorbital canal, then the reason was recorded (e.g., globe is too rostral and lateral to hit through the canal).

**Figure 11:**
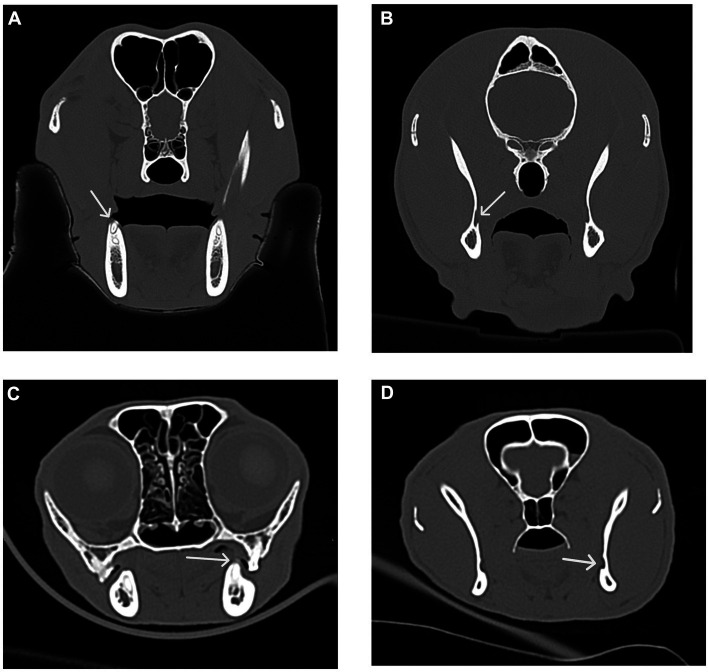
Transverse CT images were used to measure the distance between the distal crown of the mandibular third molar tooth in dogs and the mandibular first molar tooth in cats to the mandibular foramen. **(A)** Transverse CT image showing the distal crown of the right mandibular third molar tooth (white arrow) in a dog. **(B)** Transverse CT image showing the right mandibular foramen (white arrow) in a dog as determined by the first transverse slice with an open mandibular foramen. **(C)** Transverse CT image showing the distal crown of the left mandibular first molar tooth (white arrow) in a cat. **(D)** Transverse CT image showing the left mandibular foramen (white arrow) in a cat as determined by the first transverse slice with an open mandibular foramen.

**Figure 12:**
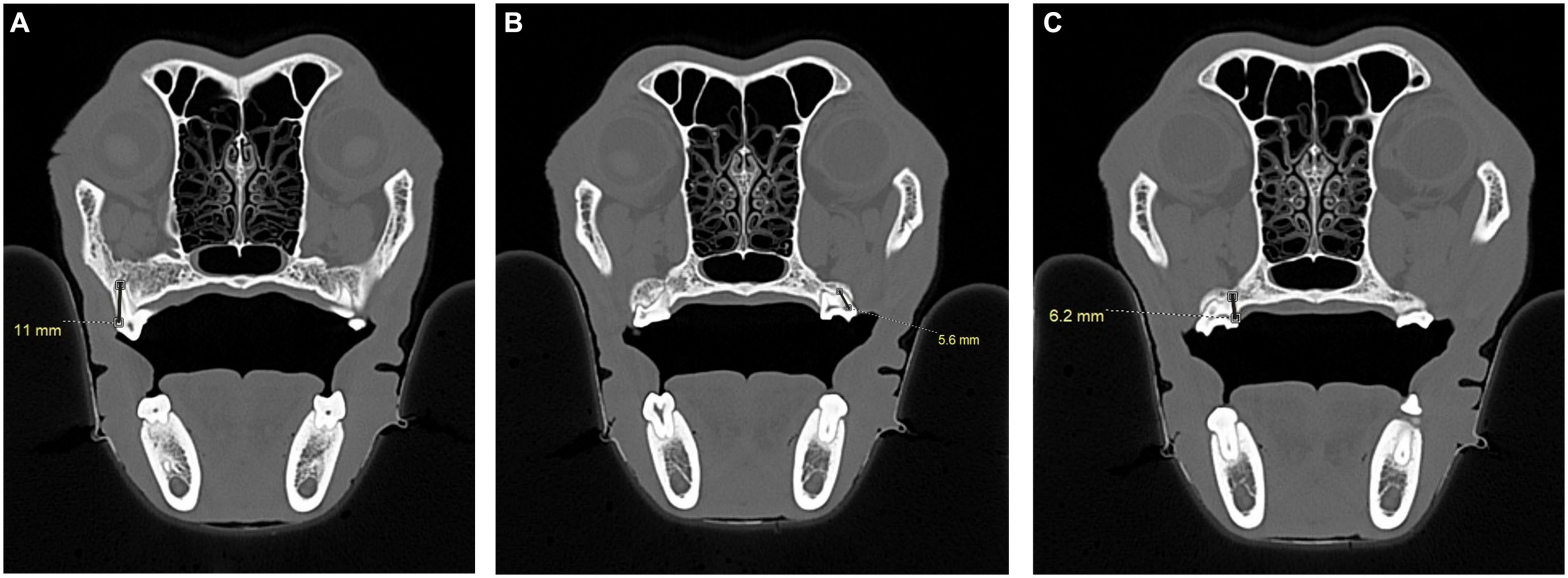
CT images demonstrating the measurement, in millimeters, of the distal buccal root length of the maxillary first molar tooth and maxillary second molar tooth in a dog. **(A)** Transverse CT image showing the measurement of the distal buccal root of the right maxillary first molar tooth in a dog. The measurement was taken from the cementoenamel junction to the root apex (black line). **(B)** Transverse CT image showing the measurement of the distal buccal root of the left maxillary second molar tooth in a dog. The measurement was taken from the horizontal pulp chamber of the crown to the root apex. **(C)** Transverse CT image of a dog skull showing the alternative measurement of the palatal root of the right maxillary second molar tooth when the entirety of the distal buccal root was not visible on a single slice. Measurements were taken from the horizontal pulp to the root apex (black line).

To simulate accidental globe puncture during the intraoral caudal maxillary nerve block, measurements were taken from the caudal border of the palate, immediately caudal to the maxillary second molar tooth in dogs and maxillary first molar tooth in cats, to the closest surface of the globe (palate-to-globe) using sagittal and transverse views (Figures 10C,D). In dogs and cats with a last mandibular molar tooth, the distance from the distal crown surface of the last mandibular molar tooth to the first CT slice with an open mandibular foramen was recorded using the transverse view (Figures 11A–D). Accurate distance measurements from the last mandibular molar tooth to the mandibular foramen were not possible on the sagittal view due to the angulation of the mandible. Root length determination for the distal buccal root of the maxillary first molar tooth in the dogs was measured from the cementoenamel junction to the root apex (Figure 12A). Root length determination for the distal buccal root of the maxillary second molar tooth was measured from the horizontal pulp horn to the root apex (Figure 12B). The cementoenamel junction was not as discernible on the maxillary second molar tooth of the dog, so the horizontal pulp horn was used instead. If the distal buccal root length of the maxillary second molar tooth was not visible on a single transverse slice, then the palatal root was measured instead using the technique described in the previous sentence (Figure 12C). All measurements were collected and recorded separately for the left and right sides. Results were grouped and placed in tables according to their use in clinical practice. Minimum and mean distance values of the infraorbital foramen-to-globe, left and right maxillary first and second molar tooth root apex-to-globe, and palate-to-globe distance values in dogs were grouped (Table 1). Minimum and mean infraorbital foramen-to-globe, left and right maxillary fourth premolar tooth root apex-to-globe, and palate-to-globe distance values in cats were grouped (Table 2). Values ≤2.8 mm were highlighted in gray. Minimum, mean, and standard deviation for infraorbital canal length, infraorbital foramen width and height, and percent of animals with a bifurcation of the infraorbital canal for all cats and dogs were grouped (Table 3). Mean distances from the distal crown surface of the last mandibular molar tooth to the mandibular foramen, and palate-to-maxillary foramen for all dogs and cats were grouped (Table 4). The percentage of distal buccal roots fused with the palatal root, and root length measurements for the maxillary first and second molar teeth in dogs were grouped (Table 5). The percentage of teeth in which the globe was centered over was determined for each body weight and skull type (Table 6), with the first and second highest percentage points highlighted in gray. Results of the most likely position of the eye were recorded for 28 dolichocephalic dogs ≤10 kg, 43 dolichocephalic dogs ≥11 kg, 56 mesocephalic dogs ≤5 kg, 56 mesocephalic dogs of 6–10 kg, 64 mesocephalic dogs ≥11 kg, 62 brachycephalic dogs ≤10 kg, and 62 brachycephalic dogs ≥11 kg (Table 6). Results were recorded for 62 MCat and 16 BCat cat eyes (Table 6). Mean, median, mode, minimum, maximum, and standard deviation of the globe diameter for all cats and dogs were grouped (Table 7). Results were calculated for 28 dolichocephalic dogs ≤10 kg, 43 dolichocephalic dogs ≥11 kg, 63 mesocephalic dogs ≤5 kg, 60 mesocephalic dogs of 6–10 kg, 64 mesocephalic dogs ≥11 kg, 62 brachycephalic dogs ≤10 kg dog eyes, 62 brachycephalic dogs ≥11 kg dog eyes, and 62 MCat and 16 BCat cat eyes. Minimum and mean infraorbital canal length, infraorbital foramen-to-globe, and recommended safe catheter or needle insertion length, for the infraorbital and modified infraorbital approach to the maxillary nerve blocks, were calculated for all cats and dogs (Table 8). All dogs below the mean infraorbital length standard deviation value for infraorbital canal length were considered “extreme brachycephalic” by the authors and included French bulldogs and pugs (four animals in total). The authors observed that French bulldogs, pugs, and Boston terriers had similarly shorter infraorbital canal mean lengths of 2–9 mm, across both ≤10 kg and ≥11 kg brachycephalic weight categories. Boston terriers, French bulldogs, and pugs were pooled together in a group labeled “extreme brachycephalic” for a total of 20 animals (14 French bulldogs, 2 pugs, and 4 Boston terriers), and their values are reported in the second-to-last row of Table 8. Minimum and mean infraorbital canal length and infraorbital foramen-to-globe length distances were recalculated for the brachycephalic dogs ≥11 kg dog group, with extreme brachycephalic dogs removed, to reflect a more accurate representation of the infraorbital canal lengths in these groups (Table 8). Groups where both the minimum infraorbital canal length and infraorbital foramen-to-globe distances were less than 10 mm were highlighted in gray to indicate that extreme caution should be used.

**Table 1:** Mean and minimum distances, in mm, from the infraorbital foramen to closest surface of the globe, from the closest root apex of the right and left maxillary first molar tooth to the outer globe, from the closest root apex of the right and left maxillary second molar tooth to the outer globe and from the end of the caudal border of the palate to the closest surface of the globe (palate-to-globe), as measured immediately caudal to the right and left maxillary second molar tooth in: mesocephalic dogs of ≤5, 6–10, and ≥11 kg, brachycephalic dogs of ≤10, and ≥11 kg, and dolichocephalic dogs of ≤10 and ≥11 kg.

Skull type and body weight	Mean and minimum distances from the infraorbital foramen to the outer globe		Maxillary first molar tooth root apex to outer globe		Maxillary second molar tooth root apex to outer globe		Palate-to-globe	
	Mean ± SD	Minimum	Mean ± SD	Minimum	Mean ± SD	Minimum	Mean ± SD	Minimum
Mesocephalic dogs, ≤5 kg***	15 ± 3.9	6.3	7.4 ± 2.6	2.1	9.8 ± 2.2	5.5	11.6 ± 2.5	6.2
Mesocephalic dogs, 6–10 kg***	22.1 ± 3.9	15	8.3 ± 2.4	6.9	13.9 ± 2.9	8.5	16.4 ± 3.4	5.3
Mesocephalic dogs, ≥11 kg	38.4 ± 6	26	23.1 ± 4.1	15	24.5 ± 4.4	16	29.7 ± 5.4	19
Brachycephalic dogs, ≤10 kg	18.3 ± 4.1	12	14.8 ± 4.7	6.2	14.8 ± 4.7	6.2	18.3 ± 4.8	9.2
Brachycephalic dogs, ≥11 kg	28 ± 9.4	17	23.9 ± 4.1	21	28.7 ± 5	16	32.1 ± 5.3	18
Dolichocephalic dogs, ≤10 kg	21.3 ± 2.3	18	8.7 ± 1.3	6.5	10.1 ± 2.2	7.7	12.2 ± 2.0	8.8
Dolichocephalic dogs, ≥11 kg	40 ± 7.0	21	21.3 ± 6.3	13	22.2 ± 6.1	14	22 ± 9	8.8

Categories with a value ≤5.5 mm have been marked with the symbol “***” to indicate that extra caution should be used to avoid ocular trauma. Specific values ≤5.5 mm have been highlighted in gray.

**Table 2:** Mean and minimum shortest distances, in mm, from the infraorbital foramen to the globe, from the apex of the distal, mesial buccal and mesial palatal roots of the right and left maxillary fourth premolar tooth to the globe, and the end of the hard palate to the globe, as measured immediately caudal to the right and left maxillary molar tooth, in mesocephalic and brachycephalic cats.

Anatomic skull measurement	Statistic	Mesocephalic cats	Brachycephalic cats
Infraorbital canal foramen to globe	Mean ± SD	8.5 ± 1.3	6.9 ± 1.1
	Minimum	5.9	4.5
Maxillary fourth premolar tooth distal root apex to globe	Mean ± SD	4.7 ± 1.1	5.1 ± 1.4
	Minimum	2.5	2.8
Maxillary fourth premolar tooth mesial palatal root apex to globe	Mean ± SD	5.8 ± 6.3	4.8 ± 1.7
	Minimum	3.7	1.6
Maxillary fourth premolar tooth mesial buccal root apex to globe	Mean ± SD	6.3 ± 1.2	5.5 ± 1.6
	Minimum	4.4	2.2
Edge of the hard palate to outer globe	Mean ± SD	8.9 ± 1.6	9.3 ± 1.7
	Minimum	5.6	6.2

Values ≤2.8 mm are highlighted in gray.

**Table 3:** Minimum (Min), Mean and standard deviation (SD) measurements describing the infraorbital canal (IOC) length, width and height at the foramen, and percent of animals with bifurcated IOC in: mesocephalic dogs ≤5 kg, mesocephalic dogs of 6–10 kg, mesocephalic dogs ≥11 kg, brachycephalic dogs ≤10 kg, brachycephalic dogs ≥11 kg, dolichocephalic dogs ≤10 kg, dolichocephalic dogs ≥ 11kg, and mesocephalic and brachycephalic cats.

Skull type and body weight	Infraorbital canal length		Infraorbital foramen width		Infraorbital foramen height		% Bifurcated IOC
	Min	Mean ± SD	Min	Mean ± SD	Min	Mean ± SD	
Mesocephalic dogs, ≤5 kg	3	8.1 ± 2.4	1.2	2.4 ± 0.45	2.3	4.8 ± 1	1.5
Mesocephalic dogs, 6–10 kg	7	12.6 ± 2.3	1.7	2.7 ± 0.44	4.4	6.1 ± 0.6	0
Mesocephalic dogs, ≥11 kg	14	21.7 ± 3.5	2.4	3.5 ± 0.77	6.1	8.3 ± 1.1	0
Brachycephalic dogs, ≤10 kg	3	6.8 ± 2.6	1.2	3.8 ± 1.6	2.5	5.4 ± 1.4	0
Brachycephalic dogs, ≥11 kg	1	10.8 ± 6.0	2.8	4.9 ± 1.3	5.1	8.2 ± 1.5	3.2
Dolichocephalic dogs, ≤10 kg	9	11.9 ± 1.9	2	2.7 ± 0.5	5	5.9 ± 0.6	0
Dolichocephalic dogs, ≥11 kg	15	23.8 ± 4.4	2	3.1 ± 0.8	5.4	8.1 ± 1.3	0
Mesocephalic cats	1	3 ± 0.9	1.8	3.1 ± 0.5	3.3	4.7 ± 0.8	0
Brachycephalic cats	1	2 ± 0.6	1.5	2.6 ± 0.4	3.5	4.5 ± 0.6	20

**Table 4:** Mean distances, in mm, from the distal crown surface of the right and left mandibular third molar tooth in dogs and mandibular first molar tooth in cats to the opening of the mandibular foramen, and from the caudal palatal border, immediately caudal to the left and right maxillary second molar tooth in dogs and maxillary first molar tooth in cats to the maxillary foramen in: mesocephalic dogs ≤5 kg, mesocephalic dogs of 6–10 kg, mesocephalic dogs ≥11 kg, brachycephalic dogs ≤10 kg, brachycephalic dogs ≥11 kg, dolichocephalic dogs ≤10 kg, dolichocephalic dogs ≥11 kg, and mesocephalic and brachycephalic cats.

Skull type and body weight	Mean distance from the left and right mandibular third molar tooth in dogs and mandibular first molar tooth in cats, to the opening of the mandibular foramen			Mean distance from the caudal palatal border, immediately caudal to the maxillary second molar tooth in dogs and the maxillary first molar tooth in cats, to the maxillary foramen		
	Mean ± SD	Minimum	Maximum	Mean ± SD	Minimum	Maximum
Mesocephalic dogs, ≤5 kg	10 ± 1.4	8	14	15 ± 2.4	8	22
Mesocephalic dogs, 6–10 kg	14 ± 2.1	9	18	17 ± 2	9	20
Mesocephalic dogs, ≥11 kg	22 ± 3.3	12	28	21 ± 2.4	16	26
Brachycephalic dogs, ≤10 kg	12 ± 2.3	8	18	17 ± 2.7	12	22
Brachycephalic dogs, ≥11 kg	18 ± 4.5	10	32	22 ± 2.8	15	28
Dolichocephalic dogs, ≤10 kg	11 ± 2	8	15	14 ± 1.7	11	17
Dolichocephalic dogs, ≥11 kg	19 ± 0.8	22	24	19 ± 4	14	32
Mesocephalic cats	15 ± 1.6	11	19	16 ± 1.6	11	20
Brachycephalic cats	12 ± 2.2	7	14	15 ± 1.7	12	17

**Table 5:** Percentage of dogs with distal buccal root fused partially or fully with the palatal root in left and right maxillary first and second molar teeth, and mean distal buccal root length of the left and right maxillary first and second molar teeth in mesocephalic dogs ≤5 kg, mesocephalic dogs of 6–10 kg, mesocephalic dogs ≥11 kg, brachycephalic dogs ≤10 kg, brachycephalic dogs ≥11 kg, dolichocephalic dogs ≤ 10 kg, and dolichocephalic dogs ≥ 11 kg.

Skull type and body weight	Percent of root fusion of the maxillary first molar tooth	Maxillary first molar tooth mean distal buccal root length	Percent of root fusion of the maxillary second molar tooth	Maxillary second molar tooth mean distal buccal/palatal root length
Mesocephalic dogs, ≤5 kg	0	6.3	67	3
Mesocephalic dogs, 6–10 kg	4	8.3	47	4
Mesocephalic dogs, ≥11 kg	5	12.4	73	7.4
Brachycephalic dogs, ≤10 kg	3	6.7	52	3.6
Brachycephalic dogs, ≥11 kg	0	9.6	53	5.1
Dolichocephalic dogs, ≤10 kg	0	7.2	50	3.3
Dolichocephalic dogs, ≥11 kg	7	11.8	84	5.6

**Table 6:** Center of the globe located over the corresponding maxillary tooth, listed by percent, in mesocephalic dogs ≤5 kg, mesocephalic dogs of 6–10 kg, mesocephalic dogs ≥11 kg, brachycephalic dogs ≤10 kg, brachycephalic dogs ≥11 kg, dolichocephalic dogs ≤ 10 kg, dolichocephalic dogs ≥11 kg, and mesocephalic and brachycephalic cats.

Skull type and body weight	Maxillary tooth							
	Canine	Second premolar	Third premolar	Fourth premolar	Lateral to fourth premolar	First molar	Second molar	Caudal to second molar
Mesocephalic dogs, ≤5 kg	0	0	0	32.2	0	64.3	3.5	0
Mesocephalic dogs, 6–10 kg	0	0	0	14.3	0	42.9	42.9	0
Mesocephalic dogs, ≥11 kg	0	0	0	0	0	28.1	59.4	12.5
Brachycephalic dogs, ≤10 kg	0	3.2	22.6	58.1	0	12.9	3.2	0
Brachycephalic dogs, ≥11 kg	3.3	22.6	0	32.3	3.2	25.8	12.9	0
Dolichocephalic dogs, ≤10 kg	0	0	0	0	0	50	35.7	14.3
Dolichocephalic dogs, ≥11 kg	0	0	0	0	0	91	27.3	63.6
Mesocephalic cats	0	0	0	45.1	0	54.5	–	–
Brachycephalic cats	0	0	0	80	–	10	–	10

The first and second highest values for each category are highlighted in gray to indicate the most likely position of the eye.

**Table 7:** Mean, median, mode, minimum, maximum, and standard deviation (SD) values for Globe diameter, listed in mm, as measured on transverse CT view, for mesocephalic dogs ≤5 kg, mesocephalic dogs of 6–10 kg, mesocephalic dogs ≥11 kg, brachycephalic dogs ≤10 kg, brachycephalic dogs ≥11 kg, dolichocephalic dogs ≤ 10 kg, dolichocephalic dogs ≥11 kg, and mesocephalic and brachycephalic cats.

Skull type and body weight	Globe diameter				
	Mean ± SD	Median	Mode	Minimum	Maximum
Mesocephalic dogs, ≤5 kg	19.8 ± 0.97	20	20	17	21
Mesocephalic dogs, 6–10 kg	21.3 ± 0.82	21	22	20	23
Mesocephalic dogs, ≥11 kg	22.9 ± 0.89	23	23	19	24
Brachycephalic dogs, ≤10 kg	21.5 ± 1.25	22	22	18	23
Brachycephalic dogs, ≥11 kg	22.8 ± 0.74	23	23	21	24
Dolichocephalic dogs, ≤10 kg	21.1 ± 0.50	21	21	20	22
Dolichocephalic dogs, ≥11 kg	23.2 ± 1.19	23	23	20	24
Mesocephalic cats	22.3 ± 0.59	22	22	21	23
Brachycephalic cats	22.3 ± 1.03	22	22	20	24

**Table 8:** Comparison of minimum and mean distances, in mm, of the infraorbital canal length and distance from the infraorbital foramen to the closest surface of the globe. Recommended safe infraorbital canal needle and catheter insertion lengths, in mm, in all dogs and cats. The second-to-last row shows extreme brachycephalic dogs (Boston terriers, French bulldogs, and pugs) weighing 7.3–18.6 kg. The last row shows brachycephalic dogs ≥11 kg with the extreme brachycephalic dogs removed.

Skull type and body weight	Mean and minimum infraorbital canal length, in mm		Mean and minimum distances, in mm, from the infraorbital foramen to the outer globe		Recommended safe needle, or catheter insertion length, in mm, for modified maxillary nerve and infraorbital nerve blocks
	Min	Mean	Min	Mean	
Mesocephalic dogs, ≤5 kg	3	8.1	6.3	15	3
Mesocephalic dogs, 6–10 kg	7	12.6	15	22.1	12
Mesocephalic dogs, ≥11 kg	14	21.7	26	38.4	14
Brachycephalic dogs, ≤10 kg	3	6.8	12	18.3	3
Brachycephalic dogs, ≥11 kg	1	10.8	17	28	See below
Dolichocephalic dogs, ≤10 kg	9	11.9	18	21.3	9
Dolichocephalic dogs, ≥11 kg	15	23.8	21	40	15
Mesocephalic cats	1	3	5.9	8.5	1
Brachycephalic cats	1	2	4.5	6.9	1
Extreme brachycephalic dogs (Boston terrier, French bulldog, and pug)	1	5.2	17	19	1
Brachycephalic dogs ≥11 kg, with extreme brachycephalic dogs removed	7	14.2	22	30.8	7–14

Groups where both the minimum infraorbital canal length and infraorbital foramen-to-globe distances are less than 10 mm are highlighted in gray to indicate that extreme caution should be used.

Percentages for when accidental globe puncture through the infraorbital canal was and was not possible were calculated for 30 dolichocephalic dogs ≤10 kg, 43 dolichocephalic dogs ≥11 kg, 59 mesocephalic dogs ≤5 kg, 49 mesocephalic dogs of 6–10 kg, 26 mesocephalic dogs ≥11 kg, 25 brachycephalic dogs ≤10 kg, and 28 brachycephalic dogs ≥11 kg, and 62 MCats and 19 BCats. These results were recorded (Table 9) along with mean, minimum, maximum, and standard deviation for palate-to-maxillary foramen distances for all cats and dogs.

**Table 9:** Percentage of cats and dogs where it was not and was possible to draw a straight line from the infraorbital foramen, through the infraorbital canal, to the globe due to the location of the eye being either too rostral, too lateral, or too caudal.

Skull type and body weight	Percentage of animals where it **was not** possible to draw a straight line from the infraorbital foramen to the globe	Percentage of animals where it **was** possible to draw a straight line from the infraorbital foramen to the globe	Mean, minimum, maximum, and standard deviation (SD) for distances, in mm, from the maxillary foramen to the globe		
			Mean **±** SD	Minimum	Maximum
Mesocephalic dogs, ≤5 kg	5	95	6.7 ± 1.6	3.1	10
Mesocephalic dogs, 6–10 kg	19	81	8.9 ± 1.8	4.9	13
Mesocephalic dogs, ≥11 kg	56	44	16.2 ± 3.5	8.4	26
Brachycephalic dogs, ≤10 kg	60	40	8.5 ± 2.7	4	15
Brachycephalic dogs, ≥11 kg	84	16	16.4 ± 3.8	7.8	26
Dolichocephalic dogs, ≤10 kg	0	100	8.8 ± 1.2	5.9	11
Dolichocephalic dogs, ≥11 kg	42	58	15.9 ± 3.3	8.3	23
Mesocephalic cats	0	100	4.5 ± 0.7	2.5	6.6
Brachycephalic cats	0	100	4.6 ± 1	2.4	6
Extreme brachycephalic dogs	80	20			
Brachycephalic dogs, ≥11 kg, with extreme brachycephalic dogs removed	79	21			

Extreme brachycephalic dogs (Boston terriers, French bulldogs, and pugs, 20 in total) and ≥11 kg Brachycephalic dogs with extreme brachycephalic dogs removed are included at the bottom. Mean, minimum, and maximum distances from the maxillary foramen to the closest surface of the globe are included on the right.

## Results

Minimum, mean, and standard deviation distances for infraorbital foramen-to-globe, and maxillary first and second molar tooth root apex-to-globe values were combined in Table 1 for all canine patients in all categories. Mesocephalic categories ≤5 and 6–10 kg had the shortest root apex-to-globe distances (Table 1). In mesocephalic dogs ≤5 kg, the minimum root apex-to-globe distances of the first and second molar teeth were 2.1 and 5.5 mm, respectively. In mesocephalic dogs of 6–10 kg, the minimum palate-to-globe distance was 5.3 mm. As shown in Table 1, all other values were greater than 5 mm. Mesocephalic dogs ≤5 kg and brachycephalic dogs ≤10 kg had the shortest infraorbital foramen-to-globe distance mean values of 15–18.3 mm. The mean value was more than twice the length of the minimum value for infraorbital foramen-to-globe distances in mesocephalic dogs ≤5 kg. In all other categories, the minimum value was less than 50% of the mean. This indicates there was more variation in minimum and mean infraorbital-to-globe distances in the mesocephalic dogs ≤5 kg than in any other group. Dolichocephalic dogs ≤10 kg, mesocephalic dogs of 6–10 kg, and brachycephalic dogs ≥11 kg had mean infraorbital foramen-to-globe distance values of 21–28 mm. Mesocephalic dogs ≥11 kg and dolichocephalic dogs ≥11 kg had infraorbital foramen-to-globe mean distance values from 38 to 40 mm. Mesocephalic dogs ≤ 5 kg had minimum root apex-to-globe distances of 5.5 mm or less from the globe; these values were highlighted in gray (Table 1). For all other dog categories, the mean and minimum root apex-to-globe distances for the maxillary first and second molar teeth were 6 mm or greater (Table 1). All dog categories had mean and minimum palate-to-globe distance values that were ≥10 mm, except for mesocephalic dogs ≤5 kg and 6–10 kg, in which minimum values were 6.2 and 5.3 mm, respectively. In all MCats and BCats, mean and minimum infraorbital foramen-to-globe distances were 4.5 mm or greater (Table 2).

Table 2 shows the shortest minimum distance values for all cats from the distal, mesial palatal, and mesial buccal roots apices of the maxillary fourth premolar tooth to the globe were 2.5, 1.6, and 2.2 mm, respectively, while mean values ranged from 4.7 to 6.3 mm. Table 2 also shows that the minimum, or shortest measured distances, from the infraorbital canal foramen-to-globe were 5.9 and 4.5 mm for MCats and BCats, respectively.

The shortest mean maxillary fourth premolar tooth root apex-to-globe distance was the distal root in MCats and the mesial palatal root in BCats (Table 2). The shortest minimum values for all cats for distance from the distal, mesial palatal, and mesial buccal roots apices of the maxillary fourth premolar tooth to the globe were 2.5, 1.6, and 2.2 mm, respectively, while mean values ranged from 4.7 to 6.3 mm. All other maxillary fourth premolar tooth root apex-to-globe distances had at least one mean or minimum value that was 5 mm or closer to the globe (Table 2). Mean maxillary foramen-to-globe distances in dogs and cats (Tables 1, 2) were consistent with trends in patient body weight, with the shortest palate-to-globe distance in mesocephalic dogs ≤5 kg, and in all cats.

The minimum, mean, and standard deviation values for infraorbital canal length and foramen height and width are visible in Table 3. The shortest minimum infraorbital canal lengths, measuring 1–3 mm, were in mesocephalic dogs ≤5 kg; brachycephalic dogs ≤10 and ≥11 kg; and all cats. Minimum infraorbital canal lengths were longer in all other groups (Table 3). In brachycephalic dogs ≥11 kg, the mean infraorbital canal length was 10.8 mm, with a standard deviation of 5.8 mm (Table 3). The widest infraorbital canal mean width values of 3.5 mm or greater were found in all brachycephalic dogs and mesocephalic dogs ≥11 kg. The smallest infraorbital canal foramen sizes, based on width and length, were in mesocephalic dogs ≤5 kg and BCats. At least one infraorbital canal was bifurcated in 20% of BCats, 3.2% of brachycephalic dogs ≥11 kg, and 1.5% of mesocephalic dogs ≤5 kg (Table 3).

The shortest mean distance from the last mandibular molar tooth to the mandibular foramen (molar-to-mandibular foramen) was found in mesocephalic dogs ≤5 kg (Table 4). The greatest difference in molar-to-mandibular foramen distance was between ≤5 and ≥11 kg mesocephalic dogs and was 11.8 mm. The longest mean mandibular last molar-to-mandibular foramen distance was 22 mm in mesocephalic dogs ≤11 kg. The shortest mean palate-to-maxillary foramen distance was 14 mm in dolichocephalic dogs ≤10 kg. The longest palate-to-maxillary foramen distance was 22 mm in brachycephalic dogs ≥11 kg (Table 4).

Mean distal buccal root length was combined with the percentage of distal buccal roots fused with the palatal root for the first and second maxillary molar teeth for all dogs (Table 5). Mesocephalic dogs ≥11 kg and dolichocephalic dogs ≥11 kg had the first and second longest mean distal buccal root lengths of 12.4 and 11.8 mm, respectively, for the maxillary first molar tooth (Table 5). Mesocephalic dogs ≥11 kg and dolichocephalic dogs ≥11 kg had the first and second longest mean distal buccal root lengths of 7.4 and 5.6, respectively, for the maxillary second molar tooth (Table 5). Mesocephalic dogs ≤5 kg had the shortest mean distal buccal root length for the maxillary first and second molars of 6.3 and 3 mm, respectively (Table 5). Dolichocephalic dogs ≥11 kg had the highest number of distal buccal and palatal roots fused in the maxillary first molar tooth, at 7%. Dolichocephalic dogs ≥11 kg were the second highest at 5%, followed by 4% of mesocephalic dogs 6–10 kg, and 3% of brachycephalic dogs ≤10 kg. The distal buccal root was fused with the palatal root of the maxillary second molar tooth in 84% of dolichocephalic dogs ≥11 kg; 74% of mesocephalic dogs ≥11 kg; 67% of mesocephalic dogs ≤5 kg; 53 and 52% of brachycephalic dogs ≥11 and ≤10 kg, respectively; 50% of dolichocephalic dogs ≤10 kg and 47% of mesocephalic dogs of 6–10 kg.

The percentage of eyes centered over a specific tooth is listed in Table 6. Brachycephalic dogs were the only category with eyes centered cranial to the maxillary fourth premolar tooth. All brachycephalic dogs and cats had the highest percentage of eyes centered over the maxillary fourth premolar tooth (Table 6). All other dog categories and MCats had the highest percentage of eyes centered over the maxillary first molar tooth or more caudal. The percentage of eyes centered over the maxillary fourth premolar tooth and maxillary first molar tooth for MCats were similar at 45.1 and 54.5%, respectively.

Results for ocular diameter are presented in Table 7 for all dogs and cats. The size of patients included in this study ranged from 1.3 to 75 kg. There was a maximum mean ocular diameter range difference of only 3.4 mm between all cats and dogs of all sizes. Mesocephalic and brachycephalic cats had larger mean globe diameters than all dogs weighing 10 kg or less.

The largest standard deviation of 1.25 mm for mean globe diameter was present in brachycephalic dogs ≤10 kg (Table 7). Minimum and maximum values for brachycephalic dogs ≤10 kg were 18 and 23 mm, respectively. The smallest standard deviation for ocular diameter was observed in dolichocephalic dogs ≤10 kg and MCats.

The ability to draw a straight line from the infraorbital foramen to the globe was recorded as a percentage for all cat and dog groups (Table 9). This was possible through the infraorbital canal in 100% of cats, and dolichocephalic dogs ≤10 kg, 95% of mesocephalic dogs ≤5 kg and 81% of mesocephalic dogs of 6–10 kg, 44% of mesocephalic dogs ≥11 kg, 40% brachycephalic dogs ≤10 kg, 16% of brachycephalic dogs ≥11 kg, 58% dolichocephalic dogs ≥11 kg, 20 and 21% of extreme brachycephalic and brachycephalic dogs ≥11 kg with the extreme brachycephalic dogs removed.

For patients where it was possible to draw a straight line from the infraorbital foramen to the globe, the shortest distance was recorded (Table 8). For patients where it was not possible, the reason was recorded in an Excel spreadsheet and included the eye being too rostral and dorsal, or the eye being too caudal, among other reasons.

## Discussion

The findings from this study may improve patient safety and efficacy during administration of the infraorbital, modified maxillary, and intraoral caudal maxillary nerve blocks, and enhance safety for dental extractions of teeth in the caudal maxillary regions. In addition, the results may help improve efficiency when performing caudal mandibulectomy and maxillectomy due to increased knowledge on the distances of the mandibular and maxillary foramens from easily identifiable dental structures in the mouth.

In the modified infraorbital approach to the maxillary nerve block, a 50-mm 20-gage over-the-needle catheter is inserted into the infraorbital canal toward the maxillary foramen (26) to a recommended depth of 5 mm, where bupivacaine is deposited. The findings from this study showing minimum and mean distances for infraorbital canal length, and infraorbital canal foramen-to-globe distances, may aid with advancement of the catheter to an appropriate length for proper analgesia in different patient sizes and skull types. Major differences in infraorbital canal length were found for different patient weight and skull shape categories. For example, the mean infraorbital canal length for dolichocephalic dogs weighing more than 11 kg was almost three times the mean infraorbital canal length of mesocephalic dogs weighing less than 5 kg. A dolichocephalic dog weighing more than 11 kg may need a nerve block catheter inserted 15 mm into the infraorbital canal to provide proper analgesia to the caudal maxillary tissues for a major procedure on the caudal maxilla. In comparison, an insertion length of only 3 mm is safe in a mesocephalic dog weighing less than 5 kg based on our findings. Safe lengths have been provided for all dog skull types and sizes, reducing the risk of globe trauma. In creating safe needle or catheter insertion lengths for all skull types and body weight categories, the authors of this study were conservative and used the minimum values for infraorbital canal lengths. In all cases, the mean distance from the infraorbital foramen to the globe was longer than the minimum infraorbital canal length. Therefore, using the infraorbital canal minimum length will provide room for error to prevent accidental globe puncture. Extra caution is recommended in all cats and mesocephalic dogs weighing less than 5 kg. Practitioners should also consider the body condition score. If a patient is morbidly obese, then they may belong in a smaller skull and body weight category, given their estimated lean body weight.

It is unclear why the mean measurements of feline infraorbital canal length in this study are slightly shorter than those found by LV Davis et al. We suspect the values differed because of where the infraorbital foramen and maxillary foramen were defined to start and stop, and because LV Davis et al. performed the measurements in the dorsal view rather than from transverse slices as in this study (24). Due to the short infraorbital canal length and large globe size relative to the patient’s size, we do not recommend inserting a catheter or needle into any feline patient’s infraorbital canal further than 1 mm, if at all.

Improved awareness of positional anatomy may aid in the safe extraction of the maxillary molar teeth in dogs and cats. According to the results of this study, there is a substantial risk of globe trauma during the extraction of maxillary first and second molar teeth in mesocephalic dogs weighing 10 kg or less. This is due to the proximity of the root apices to the eye, which were as close as 2.1 mm in some cases, as well as the high percentage of eyes that are centered over the maxillary molar teeth. In addition, the results from this study show that 47% or higher of all second molar teeth in dogs will have a distal buccal root that is fused with the palatal root. From the author’s experience, fusion of the distal buccal and palatal roots may aid in the extraction of the maxillary second molar tooth. Due to the short mean root length confirmed in this study and the frequent extent of periodontal disease, one is often able to use the wheel and axle technique to slowly rotate the un-sectioned maxillary second molar tooth out. There were no maxillary molar teeth with a mesial buccal root fused with the palatal root. Fusion of the distal buccal and palatal roots explains why the mesial root of the maxillary second molar tooth is more likely to fracture during extraction. The fractured mesial buccal root may be technically challenging for less experienced veterinarians when the maxillary first molar tooth is present. The first molar tooth limits visualization of the mesial buccal root of the second molar tooth, increasing extraction difficulty and potentially putting the patient at higher risk for globe trauma. The results of this study help to explain why sectioning the maxillary first molar tooth in dogs occasionally results in incomplete separation of the distal buccal and palatal roots, despite using proper technique. Our findings revealed large differences in root length between the maxillary first and second molar teeth, with the roots of the first molar tooth sometimes double the length of the second molar tooth. We reported that the longest maxillary first molar tooth distal buccal root lengths occurred in mesocephalic and dolichocephalic dogs weighing more than 11 kg. These results indicate that veterinarians with little dental experience may encounter more difficulty during the extraction of the maxillary first molar tooth in larger mesocephalic and dolichocephalic dogs.

The mean mandibular molar-to-mandibular foramen and palate-to-maxillary foramen distances found in this study may provide helpful information for surgical planning, accurate needle placement for nerve block, and more efficient ligation of the mandibular and maxillary arteries during surgery. Dental specialists frequently have access to standard or cone-beam CT imaging. However, when CT imaging is not available during the surgical planning stage, mean mandibular molar-to-foramen distances for all cat and dog categories may be useful when determining margins and avoiding unnecessary hemorrhage during caudal mandibulectomy and ligation of the mandibular artery during extended subtotal mandibulectomy (23). Similarly, knowledge of mean palate-to-maxillary foramen distances for all dog and cat categories may aid in surgical planning or pre-ligation of the maxillary artery prior to caudal maxillectomy to reduce major hemorrhage, a common complication (7, 27). The shortest mean mandibular molar-to-mandibular foramen and palate-to-maxillary foramen distances were observed in dolichocephalic dogs weighing less than 5 kg. This was an unexpected finding due to the long nose of dolichocephalic dogs and highlights the importance of reporting the findings of this study.

The authors of this study examined the percentage of patient skull and body weights where accidental puncture of the globe during an infraorbital nerve block was possible. The infraorbital foramen-to-globe distances were recorded to simulate the placement of a long straight 27 g nerve block needle into the infraorbital canal, during an infraorbital nerve block, to the closest globe surface. In almost all cases, the shortest infraorbital foramen-to-globe distance was acquired by angling the needle upward at an approximate 30–45° angle from the hard palate, due to the curvature of the globe. Interestingly, four of the five brachycephalic dogs ≥11 kg in which it was possible to hit the globe through the infraorbital canal (16%), had long mean infraorbital foramen-to-globe distances (22–42 mm) and long mean infraorbital canal lengths (≥9 mm), except for one French bulldog, with a short mean infraorbital canal length of 2–3 mm. However, in the French bulldog with the short mean infraorbital canal length, the infraorbital foramen-to-globe distance was a lengthy 17 mm, and an extreme dorsal angulation was needed to contact the globe due to its rostral dorsal location. Extreme dorsal angulation of a nerve block needle is unlikely to be used in clinical practice. This demonstrates that in 16% of brachycephalic dogs ≥11 kg, in which it is possible to puncture the globe through the infraorbital canal, the clinical risk of this happening is quite low because the mean infraorbital foramen-to-globe length in those dogs was so long. In the remaining 85% of brachycephalic dogs ≥11 kg, it was not possible to draw a straight line from the infraorbital foramen to the globe, indicating that globe puncture through the infraorbital canal was not possible for 84% of large brachycephalic dogs, weighing more than, based on CT imaging. Similar results were found in brachycephalic dogs ≤10 kg with slightly higher ability for globe puncture of 40%. In approximately 50% of dolichocephalic dogs ≥11 kg and mesocephalic dogs ≥11, accidental globe puncture was possible. However, the minimum lengths from the infraorbital canal foramen-to-globe were 21 and 26 mm, respectively, putting these groups at low risk clinically. Mesocephalic dogs 6–10 and dolichocephalic dogs ≤10 kg were at moderate risk. High-risk groups were mesocephalic dogs ≤5 kg, and all cats because the short mean and minimum infraorbital canal length and short infraorbital foramen-to-globe distances make accidental globe puncture through the infraorbital canal likely to happen in clinical practice. Some of the results were somewhat surprising. It was the author’s general clinical impression that all brachycephalic dogs were at high risk for globe puncture during the infraorbital nerve block due to their short infraorbital canal length. However, because there is a small percentage of brachycephalic dogs where it is possible to hit the globe through the infraorbital canal, 16% of large and 40% of small brachycephalic dogs, we have made our safe recommendations for infraorbital needle insertion depths for all animals based on the minimum canal lengths encountered. Previous studies have shown that virtual anatomical bone measurements taken from CT imaging are reliable and accurate (28). However, the effects of rotational changes in patient positioning on the accuracy of feline and canine anatomical measurements taken from CT images were not examined in this study. Changes in human patient positioning about the x-, y-, and z-axis, otherwise known as roll, pitch, and yaw, have been shown to significantly affect anatomical measurements taken by CT (29, 30). All patients included in this study were scanned in sternal recumbency. It is unknown how minor changes in patient positioning, such as the head tipped slightly to one side, may have affected the accuracy of the measurements collected in this study and should be considered a limitation. This study reports which maxillary tooth the globe is centered over, by percent, providing valuable clinical information on which maxillary teeth veterinarians should be most cautious extracting for different body and skull types. When elevating the maxillary fourth premolar teeth, the most caution should be used in all cats, all brachycephalic dogs, and mesocephalic dogs weighing 5 kg or less. When elevating the maxillary molar tooth, the most caution should be used in all cats, all mesocephalic and dolichocephalic dogs, and brachycephalic dogs weighing more than 11 kg. In addition to these findings, caution should be used when elevating the maxillary second and third premolar teeth in any brachycephalic dog, particularly extreme brachycephalic dogs with a rostrally placed eye.

Useful minimum and mean ranges have been provided in this study and may be used by veterinarians to make safe clinical decisions during caudal maxillary nerve blocks and the extraction of the maxillary fourth premolar tooth and maxillary molar teeth for different weight and skull types in canine and feline patients. In feline patients, the root apices of the maxillary fourth premolar tooth were extremely close to the globe in some cats. Similarly, the maxillary first and second molar tooth root apices were extremely close to the globe in some dogs. Based on the minimum root-apex-to-globe results found in this study, extreme caution should be used when performing maxillary first and second molar tooth extractions in mesocephalic dogs ≤5 kg and intraoral caudal maxillary blocks in mesocephalic dogs of 6–10 kg. Iatrogenic globe penetration frequently has devastating consequences for the patient, with most animals requiring enucleation (2, 9, 15, 19).

The authors recommend that the more conservative minimum values be used, rather than mean values, for each category. Clinically, achieving absolute precision in the insertion depth of a dental elevator or nerve block needle is difficult or impossible, given the small difference of a few millimeters. For any patient category with a distance of ≤10 mm to the globe for either infraorbital, intraoral maxillary nerve blocks or tooth extraction, extreme care should be taken not to exceed the minimum values. Multiple reports of ocular trauma incurred during dental extractions have been reported in cats and dogs (14, 21, 22). The results of this study show that the globe is centered over the maxillary fourth premolar tooth and first molar tooth in 100% of MCats and 90% of BCats. The close proximity of the caudal maxillary tooth roots to the globe leaves very little room for error and demonstrates that the most caution should be used while extracting the maxillary fourth premolar tooth in cats and maxillary molar teeth in dogs. Extraction of these teeth can be performed safely using a short finger stop at the tip of the dental instrument, using correctly sized dental instruments, and being particularly cautious when periodontal disease has eroded the regional bone. In addition to these findings, the minimum palate-to-globe distances were small at 5.6 and 6.2 mm in MCats and BCats. This information is useful for those practitioners who perform intraoral maxillary blocks immediately caudal to the maxillary molar tooth in cats (10).

## Conclusion

Our results provide useful clinical information that will enable general practitioners, along with veterinary dentistry specialists, to make safer decisions when providing analgesia in the oral cavity and nasal regions and when performing dental extractions involving the maxillary fourth premolar tooth and maxillary first molar tooth in cats and dogs and the maxillary second molar tooth in dogs. In conclusion, the most caution should be used when performing infraorbital and maxillary nerve blocks in mesocephalic and dolichocephalic dogs weighing less than 10 kg and all cats. The deep caudal maxillary nerve block should not be used on cats and small dogs. The maxillary nerve block using the modified infraorbital approach with the author’s recommended safe needle and catheter insertion lengths should provide safe and effective analgesia administration. Mean measurements for distances from the mandibular molar tooth distal crown to the mandibular foramen (mandibular third molar tooth in dogs and mandibular first molar tooth in cats), and palate-to-maxillary foramen, may be used for surgical planning and ligation for hemostasis for caudal mandibulectomies and maxillectomies, particularly when CT imaging is not available.

Our recommendations for future research are to investigate how rotational changes in patient positioning about the x-, y-, and z-axis affect the accuracy of anatomical CT measurements on canine and feline skulls. In addition, we recommend a cadaver study using the same skull and body weight categories established in this study to confirm the accuracy and safety of our findings for safe needle or catheter insertion lengths. And finally, to test the clinical usefulness of the mean last molar tooth to the mandibular foramen or palate-to-maxillary foramen distances in simulated caudal mandibulectomies and maxillectomies in cadavers.

## Data Availability

The raw data supporting the conclusions of this article will be made available by the authors, without undue reservation.
